# Differential effects of simvastatin on IL-13-induced cytokine gene expression in primary mouse tracheal epithelial cells

**DOI:** 10.1186/1465-9921-13-38

**Published:** 2012-05-14

**Authors:** Amir A Zeki, Phil Thai, Nicholas J Kenyon, Reen Wu

**Affiliations:** 1 U.C. Davis, School of Medicine, U.C. Davis Medical Center, Department of Internal Medicine, Division of Pulmonary, Critical Care, and Sleep Medicine, Center for Comparative Respiratory Biology & Medicine, Davis, CA, USA; 2 Genomics & Biomedical Sciences Facility (GBSF), 451 Health Sciences Drive, Suite #6510, Davis, CA, 95616, USA

**Keywords:** Statin, Asthma, Mevalonate pathway, Airway epithelium, HMG-CoA reductase, Cytokines, Chemokines, Gene expression, Mouse tracheal epithelium

## Abstract

**Background:**

Asthma causes significant morbidity worldwide in adults and children alike, and incurs large healthcare costs. The statin drugs, which treat hyperlipidemia and cardiovascular diseases, have pleiotropic effects beyond lowering cholesterol, including immunomodulatory, anti-inflammatory, and anti-fibrotic properties which may benefit lung health. Using an allergic mouse model of asthma, we previously demonstrated a benefit of statins in reducing peribronchiolar eosinophilic inflammation, airway hyperreactivity, goblet cell hyperplasia, and lung IL-4 and IL-13 production.

**Objectives:**

In this study, we evaluated whether simvastatin inhibits IL-13-induced pro-inflammatory gene expression of asthma-related cytokines in well-differentiated primary mouse tracheal epithelial (MTE) cell cultures. We hypothesized that simvastatin reduces the expression of IL-13-inducible genes in MTE cells.

**Methods:**

We harvested tracheal epithelial cells from naïve BALB/c mice, grew them under air-liquid interface (ALI) cell culture conditions, then assessed IL-13-induced gene expression in MTE cells using a quantitative real-time PCR mouse gene array kit.

**Results:**

We found that simvastatin had differential effects on IL-13-mediated gene expression (inhibited eotaxin-1; MCP-1,-2,-3; and osteopontin (SPP1), while it induced caspase-1 and CCL20 (MIP-3α)) in MTE cells. For other asthma-relevant genes such as TNF, IL-4, IL-10, CCL12 (MCP-5), CCL5 (RANTES), and CCR3, there were no significant IL-13-inducible or statin effects on gene expression.

**Conclusions:**

Simvastatin modulates the gene expression of selected IL-13-inducible pro-inflammatory cytokines and chemokines in primary mouse tracheal epithelial cells. The airway epithelium may be a viable target tissue for the statin drugs. Further research is needed to assess the mechanisms of how statins modulate epithelial gene expression.

## Introduction

Asthma is a leading cause of morbidity worldwide affecting children and adults alike. Novel and innovative therapies are needed to treat this chronic condition that often progresses to irreversible airflow obstruction. There is increasing evidence that some widely-available therapies, like the statin drugs (‘statins’), may benefit patients with asthma. The statins are widely-used cholesterol-lowering drugs that have revolutionized the treatment of cardiovascular diseases. They inhibit the enzyme 3-hydroxy-3-methlyglutaryl–coenzyme A (CoA) reductase (HMGR) which converts HMG-CoA into mevalonate (MA), the rate-limiting step of cholesterol biosynthesis (i.e. the MA pathway). The statins also have pleiotropic HMGR- or MA-independent effects beyond lowering cholesterol that, at least in part, belie their immunomodulatory and anti-inflammatory properties [1,2]. Observational studies show an association between statin use and improved lung health in asthma and chronic obstructive pulmonary disease (COPD), e.g. reduced decline in FEV_1_ and FVC, reduced exacerbations, and reduced mortality in COPD [3-7] Several small clinical trials using statins to treat asthma have shown an anti-inflammatory effect as measured by reduced sputum leukotriene levels and leukocyte cell counts such as macrophages and eosinophils [8-10]. Ongoing clinical trials in asthma and COPD will determine whether statins also have meaningful clinical benefits (http://www.clinicaltrials.gov). However, the underlying mechanisms and level at which the statin benefit may occur is complex and not fully delineated.

We previously demonstrated that simvastatin ameliorates allergic eosinophilic airway inflammation, decreases IL-13 and IL-4 levels in lung lavage fluid, and improves airway hyperreactivity (AHR) in the ovalbumin mouse model of asthma [11]. We also showed that simvastatin attenuates early hallmarks of airway remodeling such as goblet cell hyperplasia, arginase expression, and arginase enzyme activity in mouse lung [12]. Others have shown similar results using different animal models of lung inflammation [13]. A small number of studies evaluating the effects of statins on human nasal, oral, and airway epithelial cells have been reported [14-22], however, the models utilized and hypotheses tested are not directly relevant to asthma. But a recent clinical trial using oral simvastatin to treat asthma, as add-on therapy to inhaled budesonide, showed an additive effect to the inhibition of sputum eosinophils [9]. However, the tissue specificity of statins in lung disease remains a question of active research, and it is not clear in what lung cell type(s) do the statins have their greatest effect. Beyond systemic effects *in vivo* or in humans*,* this anti-inflammatory effect could be at the level of the pulmonary endothelium, mesenchyme, or epithelium, if not the inflammatory cells themselves.

To explore the role of the statins in regulating airway epithelial pro-inflammatory responses relevant to human allergic asthma, and to build on our prior work in the ovalbumin mouse model, we conducted a series of *in vitro* experiments using primary mouse tracheal epithelial cells (as previously developed by our lab) [23,24]. *We hypothesized* that simvastatin inhibits the expression of IL-13-induced cytokines and chemokines in primary mouse tracheal epithelial cells. Our data indicate that simvastatin has differential effects on mouse epithelial cytokine gene expression. While it inhibited the expression of some IL-13-inducible cytokines, other genes important in inflammation and host immune responses were induced by simvastatin independent of IL-13. Our results suggest that during IL-13-mediated stimulation, simvastatin may suppress airway epithelial pro-inflammatory responses relevant to asthma pathogenesis. However, the induction of some genes by simvastatin is an interesting finding that will require exploration, as this could have important therapeutic implications for airway diseases given that a large segment of the human population takes statins.

## Methods

### Mouse tracheal epithelial cells

All mice were housed in 24-hr dark/light conditions breathing filtered air in our mouse vivarium facility at U.C. Davis. Our protocol was approved by the IACUC and monitored by on-campus veterinary scientists. With some modifications of the procedure as described in You et al and Robinson et al, we harvested primary mouse tracheal epithelial (MTE) cells from naïve Balb/c mice under sterile conditions [23,25]. Briefly, mice were sacrificed by overdose using pentobarbital, then dipped whole body (while sparing the mouth and nares) in ethanol to sterilize, followed by careful blunt dissection and removal of their lungs and tracheas. Polyethylene (PE) tubing (0.86 mm diameter) was inserted into the trachea and secured with sterile sutures. The trachea was then rinsed using D-media. Enzymatic digestion was used to remove cells from the tracheal lumen by injecting seven drops of D-media + 0.2% Pronase Mix into the trachea, followed by suture closure and briefly heating the end of the PE tubing to seal it. Tracheas were then placed in D-media and stored overnight at 4°C. Collagen matrix coating of transwells was made of 80% collagen (PureCol™, Inamed Biomaterials, Fremont, CA), 13.3% 1:1 F12 and DMEM, and 6.7% 0.2 M NaOH. At least 300 μL of collagen mix was used to thoroughly cover each transwell, and allowed to solidify over 1–2 hrs at 37°C. Tracheal epithelial cells were then isolated and cultured as follows: the PE tubing was cut off, 5 mL of media was passed through each trachea and pooled into 50 mL conical tubes (30 mL per conical tube). Cells were centrifuged at 1,000 rpm for 15 minutes, then supernatant removed to 5 mL, and pellet resuspended. For multiple tracheas, tubes were combined together and re-centrifuged for 10 minutes, then the supernatant was removed to the 5 mL volume. The cell suspension was corrected to a volume of 10 mL in D-media + 100 nM retinoic acid (RA). Cell suspensions (300 μL) were then evenly distributed into each 12-well transwell, then 1 mL D-media + RA was added to each of the lower wells in complete immersion. Tracheal epithelial cells were then allowed to adhere to the collagen matrix for 4 days. After 2 weeks, cells were removed from immersion culture and switched to C-media + RA (in biphasic, air-liquid interface (ALI) cell culture conditions) with 100 μL on top and 1 mL on bottom. Tracheal cells were then grown in ALI conditions for 4 weeks until >80-90% confluence was achieved. Two experiments were conducted successfully, and three SA Biosciences PCR array plates were used of which only two had reliable data.

All mouse primary tracheal epithelial cells grown in ALI were pre-treated with simvastatin (Sim, 10 μM) for 24 hrs, then stimulated with IL-13 (20 ng/mL) and co-incubated with Sim for 48 hrs (total Sim exposure of 72 hrs). Experiments were carried out under these drug and cytokine concentrations unless specified otherwise in the text.

### Drugs and reagents

Simvastatin (Sim) was purchased as a pure powder from Sigma-Aldrich (St. Louis, MO) and prepared as a stock solution in dimethyl sulfoxide (DMSO), then diluted to 10 μM Sim using sterile 1X PBS and/or cell culture media. Control groups of DMSO-only treatment done previously showed no effect on measured gene expression. The final concentration of DMSO used to dissolve Sim was 1:1,000 or 1:5,000 dilution using a Sim stock solution of 10 mM or 50 mM, respectively. Simvastatin was stored at −20°C and slowly heated to 37°C in a water bath before applying to cells.

### Cytokines

Interleukin-13 (IL-13) was purchased from R&D Systems and reconstituted using sterile 1X PBS or water, and stored at 4°C when not in use. IL-13 was then diluted to the appropriate dose using the cell culture medium described above and slowly heated to 37°C in a water bath before applying to cells.

### RT-PCR gene array for mouse primary tracheal epithelial cells

The PAMM-011E-4, RT [2] Profiler™ PCR Array kit for mouse inflammatory cytokines and receptors was purchased from SA Biosciences (Frederick, MD, USA). Following the manufacturer’s instructions, we used the housekeeping gene Hsp90ab1 because this had the most consistent level of expression across the four different treatment groups, with the least variability in Ct values or level of expression, when compared to other mouse housekeeping genes including GAPDH, β-Actin, Gusb, and Hprt1. Total RNA was extracted from primary MTE cells after simvastatin treatment using RNA TRIzol reagent (Invitrogen, Carlsbad, CA) according to the manufacturer’s protocol. Two micrograms of extracted RNA was converted to cDNA by adding MMLV-reverse transcriptase (Promega, Madison, WI), 5X buffer, dNTP, and oligo-dT primers in a total volume of 20 μL. The reaction was further diluted 1:5 to a total volume of 100 μL with nuclease-free water and used for real-time PCR analysis. After adding cDNA samples to the pre-coated array plates, the RT-PCR reaction was carried out in a 384-well optical PCR plate according to the manufacturer’s instructions. Results were analyzed by the ABIPRISM 7900HT Sequence Detection system (Applied Biosystems). The relative amount of mRNA in each sample was calculated by normalizing its threshold cycle (Ct) value to the Ct value of the housekeeping gene Hsp90ab1. The calculation formula used: **2**^**– [GOI(Ct) - Hsp90ab1(Ct)]**^ in arbitrary units, where GOI stands for “gene of interest.” Results are presented as gene expression relative to the housekeeping gene Hsp90ab1.

### Statistical analyses

The data are expressed as mean ± SEM. Group differences for parametric continuous data were assessed by Student’s t-test or 1-way ANOVA with Tukey’s correction for multiple comparisons using the Prism 5 software package (Graphpad, Inc., San Diego, CA). Differences were considered to be statistically significant for a 2-way *alpha* and p-value ≤ 0.05.

## Results

### Simvastatin inhibits the expression of pro-inflammatory cytokines in mouse tracheal epithelial cells

**Figure 1:**
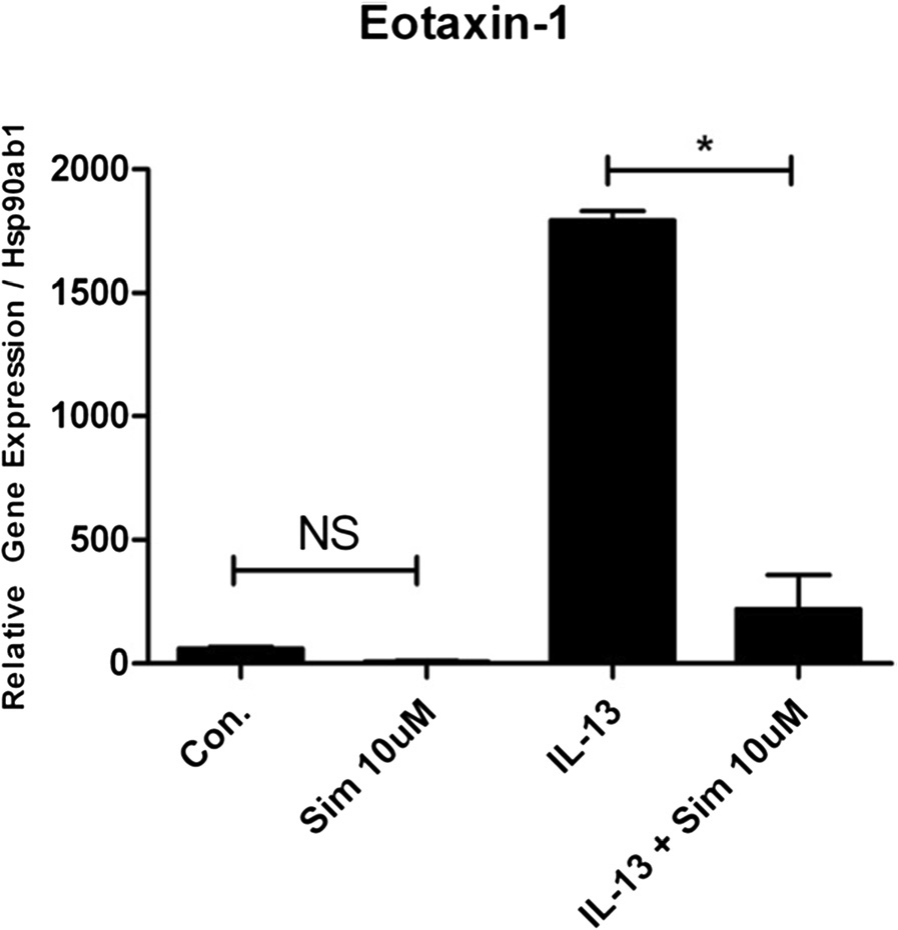
**Simvastatin inhibits Eotaxin-1 expression in mouse primary tracheal epithelial cells.** Mouse primary tracheal epithelial cells grown in air-liquid interface (ALI) were pre-treated with simvastatin (Sim, 10 μM) for 24 hrs, then stimulated with IL-13 (20 ng/mL) and co-incubated with Sim for 48 hrs (total Sim exposure of 72 hrs). Gene expression was evaluated by RT-PCR. Treatment with Sim reduced eotaxin-1 (CCL11) expression by 87.8% (*p < 0.0001 by ANOVA). Simvastatin treatment had no statistically significant effect on basal expression of eotaxin-1 (Con. versus Sim 10 μM; p = NS by ANOVA)

**Figure 2:**
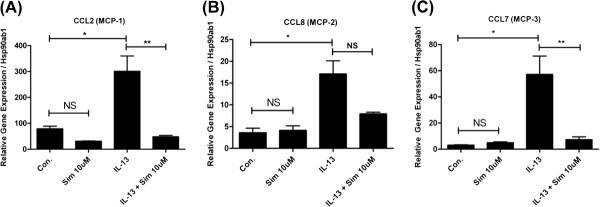
**Simvastatin inhibits Monocyte Chemotactic Protein (MCP) gene expression in mouse primary tracheal epithelial cells.** Mouse primary tracheal epithelial cells grown in air-liquid interface (ALI) were pre-treated with simvastatin (Sim, 10 μM) for 24 hrs, then stimulated with IL-13 (20 ng/mL) and co-incubated with Sim for 48 hrs (total Sim exposure of 72 hrs). Gene expression was evaluated by RT-PCR. (**A**) Sim reduced IL-13-induced MCP-1 (CCL2) expression by 84.0% (*p < 0.005 for control vs. IL-13, and **p < 0.005 for IL-13 vs. IL-13 + Sim; both by ANOVA). Simvastatin treatment had no statistically significant inhibition of basal MCP-1 expression (Con. versus Sim 10 μM; p = NS by ANOVA). (**B**) Sim reduced IL-13-induced MCP-2 (CCL8) expression by 53.7% (*p < 0.05 for control vs. IL-13, and p = NS for IL-13 vs. IL-13 + Sim by ANOVA or t-test). Simvastatin treatment did not inhibit the basal expression of MCP-2 (Con. versus Sim 10 μM; p = NS by ANOVA). (**C**) Sim reduced IL-13-induced MCP-3 (CCL7) expression by 87.2% (*p < 0.05 for control vs. IL-13, and **p < 0.05 for IL-13 vs. IL-13 + Sim; both by ANOVA). Simvastatin treatment did not inhibit the basal expression of MCP-3 (Con. versus Sim 10 μM; p = NS by ANOVA)

We explored whether simvastatin (10 μM) inhibits IL-13-induced pro-inflammatory cytokine/chemokine gene expression using a RT-PCR gene array. Treatment with Sim reduced eotaxin-1 (CCL11) expression by 87.8% (*p < 0.0001 by ANOVA) (Figure 1). Simvastatin also inhibited the expression of eotaxin-2 (CCL24) in mouse airway epithelial cells by 50.2% but this did not reach statistical significance (p = NS by ANOVA or t-test, data not shown). Simvastatin also reduced IL-13-induced MCP-1 (CCL2) expression by 84.0% (*p < 0.005 for control vs. IL-13, and **p < 0.005 for IL-13 vs. IL-13 + Sim; by ANOVA, Figure 2A). Simvastatin reduced IL-13-induced MCP-2 (CCL8) expression by 53.7% (*p < 0.05 for control vs. IL-13, and p = NS for IL-13 vs. IL- 13 + Sim by ANOVA or t-test, Figure 2B). Simvastatin reduced IL-13-induced MCP-3 (CCL7) expression by 87.2% (*p < 0.05 for control vs. IL-13, and **p < 0.05 for IL-13 vs. IL-13 + Sim; by ANOVA, Figure 2C).

The mouse genes for which there was either insufficient IL-13 stimulation and/or lack of statistically significant inhibition of gene expression by simvastatin include the following (data not shown): TNFβ, TNF, CCL9 (MIP-1γ), IL-1β, IL-4, IL-13, IL-10, IL-11, IL-15, IL-17B, CRP, CCL12 (MCP-5), CCL19 (MIP-3β), CCL5 (RANTES), CXCL1 (Gro-α), CXCL5, CXCL10 (IP-10), CXCL11, CXCL12 (SDF), C3, ABCF1, CCR1, CCR3-6, CCR8, CCR9, CXCL9, and IL-5Ra. Simvastatin also inhibited the expression of IL-13-induced TGFβ expression by 80.1% but this was not statistically significant (p = NS by ANOVA or t-test, data not shown). It is important to note that IL-13 is not known to induce some of these aforementioned cytokines/chemokines given different cell signaling pathways, and this may also reflect the lack of induction or clear differences with statin treatment.

One component of the IL-6 receptor is the IL-6 signal transducer (IL-6st) protein (a.k.a. Glycoprotein 130), which is important for signal transduction following cytokine engagement of the receptor. Treatment with Sim inhibited the IL-13-induced expression of IL-6st gene by 54% (p = NS by ANOVA, but *p = 0.039 by t-test, data not shown).

**Figure 3:**
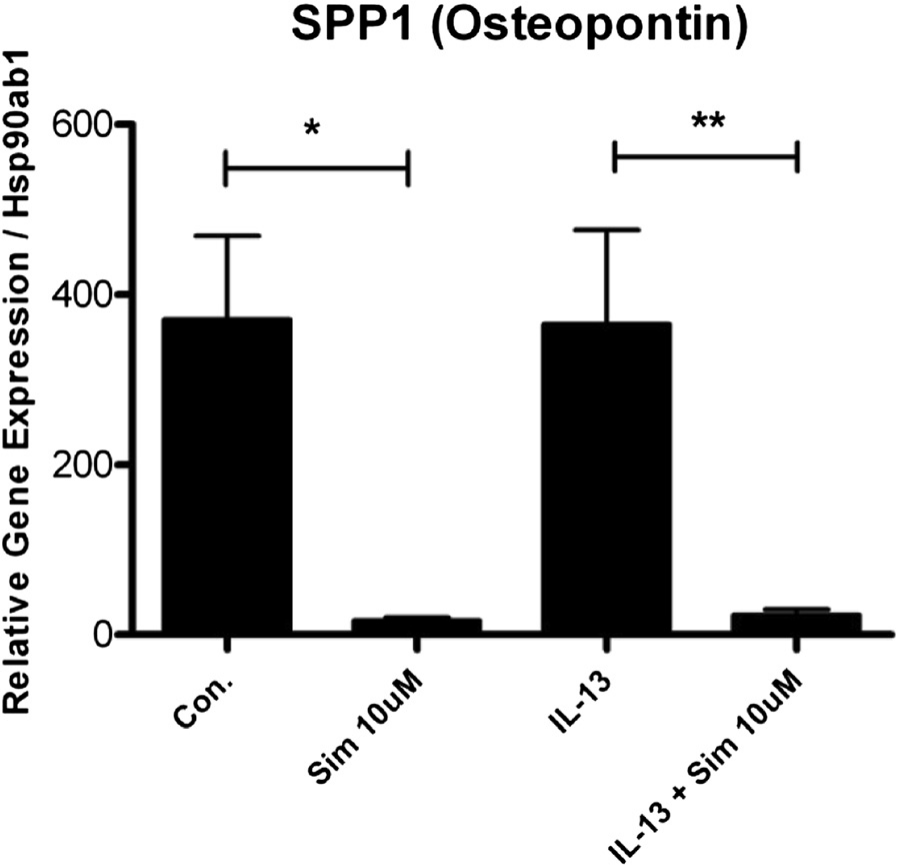
**Simvastatin inhibits the expression of Osteopontin (SPP1) in mouse primary tracheal epithelial cells.** Mouse primary tracheal epithelial cells grown in air-liquid interface (ALI) were pre-treated with simvastatin (Sim, 10 μM) for 24 hrs, then stimulated with IL-13 (20 ng/mL) and co-incubated with Sim for 48 hrs (total Sim exposure of 72 hrs). Gene expression was measured by RT-PCR. In the control group, Sim inhibited the expression of osteopontin by 95.5% (*p < 0.05 by ANOVA) independent of IL-13 stimulation, and in the IL-13 group Sim decreased osteopontin expression by 93.6% (**p < 0.05 by ANOVA)

In our evaluation of primary MTE cells, one gene in particular displayed significant inhibition with simvastatin, the pro-inflammatory and pleiotropic cytokine osteopontin or SPP1 (secreted phosphoprotein 1). In the control group, Sim inhibited the expression of osteopontin by 95.5% (*p < 0.05 by ANOVA) independent of IL-13 stimulation, and in the IL-13 group Sim decreased osteopontin expression by 93.6% (**p < 0.05 by ANOVA) (Figure 3).

### Simvastatin induces the expression of genes in mouse tracheal epithelial cells

**Figure 4:**
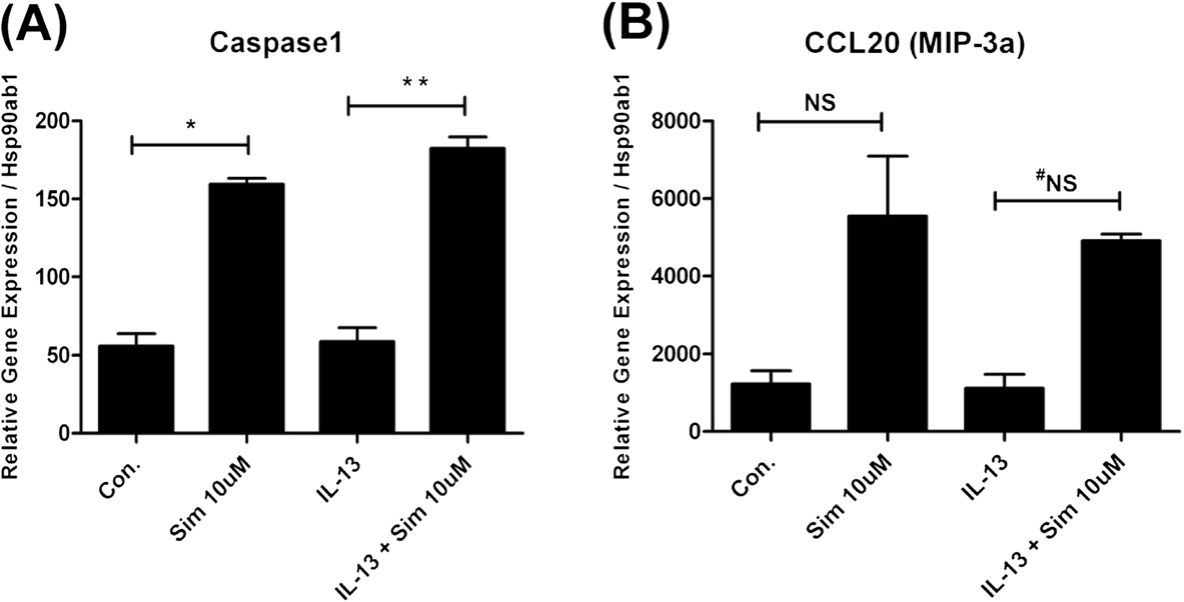
**Simvastatin induces the expression of CCL20 (MIP-3α) and Caspase-1 in mouse primary tracheal epithelial cells.** Mouse primary tracheal epithelial cells grown in air-liquid interface (ALI) were pre-treated with simvastatin (Sim, 10 μM) for 24 hrs, then stimulated with IL-13 (20 ng/mL) and co-incubated with Sim for 48 hrs (total Sim exposure of 72 hrs). Gene expression was evaluated by RT-PCR. (**A**) IL-13 did not induce caspase-1 expression. Treatment with simvastatin induced caspase-1 expression by 285% in the control group (*p < 0.005 by ANOVA) and by 310% in the IL-13 group (**p < 0.005 by ANOVA). (**B**) IL-13 did not induce CCL20 expression. Treatment with simvastatin induced CCL20 expression by 451% in the control group (p = NS by ANOVA or t-test) and by 441% in the IL-13 group (^#^p = NS by ANOVA, but p = 0.011 by t-test)

Simvastatin treatment induced the expression of a minority of genes as detected in our PCR array analysis. For the limited collection of genes we evaluated, we observed the induction of CCL20 (MIP-3α) and caspase-1 expression by simvastatin. Exposure to IL-13 did not induce caspase-1 expression, however, treatment with simvastatin induced caspase-1 expression by 285% in the control group (*p < 0.005 by ANOVA) and by 310% in the IL-13 group (**p < 0.005 by ANOVA) (Figure 4A). Also, IL-13 did not induce CCL20 expression, however, treatment with simvastatin induced CCL20 expression by 451% in the control group (p = NS by ANOVA or t-test)and by 441% in the IL-13 group (^#^p = NS by ANOVA, but p = 0.011 by t-test) (Figure 4B).

For all of the data reported, in two separate experiments we did not observe any qualitative or visual evidence of cell morphological changes, gross signs of cytotoxicity, or overt cell death with simvastatin treatment.

## Discussion

We and others previously showed that systemic treatment of mice with simvastatin attenuates allergic inflammation, decreases the production of T_h_2 cytokines IL-4 and IL-13, reduces goblet cell hyperplasia, and improves AHR [11,12,26,27]. Collectively, these results suggested both an anti-inflammatory and epithelial anti-remodeling effect of simvastatin *in vivo*[11,12,28]. Given the central role of the airway epithelium in asthma pathogenesis [29,30], we wanted to determine the effect of simvastatin on genes known to play a role in allergic airway inflammation using primary MTE cells. We focused on IL-13-responsive genes given (a) our prior *in vivo* observations showing marked simvastatin inhibition of IL-13 production in mouse lung lavage fluid [11], and (b) its importance in human asthma and allergic inflammation. Our primary findings are that *in vitro* treatment with simvastatin (1) decreased the expression of IL-13-induced genes such as eotaxin-1 and the MCPs, while (2) it enhanced the basal expression of certain IL-13 non-inducible genes. To our knowledge, this is the first paper that evaluates the effect of simvastatin on IL-13-induced pro-inflammatory gene expression in primary MTE cells using an ALI system, consistent with our previous work showing that simvastatin attenuates inflammatory pathways pertinent to eosinophilic allergic inflammation *in vivo*[11].

Our data show significant inhibition of IL-13-inducible CCL class cytokines eotaxin-1, and MCP-1,-2, and −3 by simvastatin (Figures 1 and 2). These cytokines are important eosinophil chemoattractants relevant to human allergic asthma. Eotaxins are potent eosinophil tissue chemoattractants, and the MCPs inhibit IL-12 and increase IL-4 production leading to enhanced T_h_2 responses, and monocyte, basophil, and eosinophil recruitment. Pertinent to our findings, there is emerging clinical evidence that simvastatin can reduce sputum eosinophil counts. A recent clinical study in asthmatics showed that simvastatin 10 mg (taken orally) plus inhaled budesonide resulted in a significant reduction in sputum eosinophils when compared to the budesonide control group (10.0% reduction vs. 6.75% reduction, p < 0.02 for %eosinophils) [9]. Although our current data are derived from *in vitro* experiments, it is plausible that simvastatin’s *in vivo* anti-inflammatory effects may also be due to suppression of cytokine gene expression in the airway epithelium resulting in reduced eotaxin production, and hence, reduced eosinophil tissue trafficking into airways. Candidate downstream targets of the statin effect include both the sterol and isoprenoid (non-sterol) branch pathways, i.e. cholesterol vs. Rho/Ras/Rac GTPase signaling in the MA pathway. Further work is needed to better characterize the mechanism(s) involved in the regulation of gene expression by statins in the airway epithelium.

In addition to inhibiting these important multifunctional T_h_2 cytokines, simvastatin also inhibited the basal gene expression of osteopontin (a.k.a. SPP1) independent of IL-13 (Figure 3). Osteopontin is an integrin ligand and a cytokine with a variety of actions important in asthma pathogenesis, including stimulating macrophages, recruiting neutrophils and T_h_1 cells, and promoting anti-apoptotic pathways [31]. It also induces airway mucin production and has an emerging role in the pathogenesis of both asthma and COPD [32-35]. There is significant interest in the role of osteopontin in asthma pathogenesis where Simoes and colleagues recently showed that ovalbumin-exposed mice lacking the osteopontin gene did not develop airway hyperresponsivness or remodeling [36]. Prior studies showed that osteopontin is produced by a variety of cells including bone, smooth muscle, epithelial, T lymphocyte, and dendritic cells [37]. Our results provide evidence that it is expressed by mouse tracheal epithelium and is either a marker of allergic inflammation or is a critical cytokine in disease pathogenesis. This adds to the evidence that simvastatin inhibits important pro-inflammatory cytokines relevant to airway diseases, especially the contribution of the airway epithelium to allergic T_h_2 inflammation.

Unexpectedly, simvastatin also induced the expression of CCL20 (MIP-3α) and caspase-1 (Figure 4), independent of IL-13. Although CCL20 is a pro-inflammatory cytokine that attracts natural killer (NK) cells and memory T-cells to sites of inflammation, this peptide also retains potent antimicrobial effects even greater than HBD-2 [38,39]. Caspase-1 is known for its activation of apoptosis and pro-inflammatory regulation of IL-1β [40,41], however, it may also mediate lung healing processes via IL-1β [41] and critical host defense mechanisms that prevent infections [40]. Given the pleiotropic effects of CCL20 and caspase-1 [42] it is unclear whether the induction of these genes by simvastatin would have a beneficial or harmful effect overall. This would dependent on the context of the intact/physiologic airway compartment; the presence of exogenous stimulants, the microbial environment, presence of infection, and overall immune milieu. However, we know from multiple studies using different animal models of lung inflammation, that systemic treatment with statins attenuates lung and peri-bronchiolar inflammation, reduces epithelial mucus production, and improves AHR suggesting that the sum effect in the intact host is likely beneficial [11,27,43-45].

It is well-accepted that the cytokine milieu in asthma is rather complex and involves, amongst many different immune cell types, T-cell-mediated mechanisms such as Th1, Th2, Th17 and regulatory T_reg_ pathways [46]. Interleukin-1β, CXCL10 (IP-10), and TNF are all Th1 polarized cytokines that play a role in the pathogenesis of asthma [46-48]. However, unlike CCL20 (MIP-3α) and caspase-1, simvastatin treatment did not inhibit or induce these important cytokines. Thus, our data do not support a statin effect on the *basal* gene expression of IL-1β, CXCL10 (IP-10), or TNF. Besides inhibition of eotaxin-1 and MCP expression by simvastatin, neither IL-13 nor simvastatin treatment changed the expression of other asthma-relevant Th2-type cytokines such as IL-4, CCL5 (RANTES), CCR3, or IL-5Ra. Given the central role these cytokines/chemokines and receptors play in eosinophilic trafficking, we speculate that the potent anti-eosinophilic effect of simvastatin seen previously in animal models [11,26] may not be due to epithelial effects alone, but might involve the vascular compartment or other airway resident cells that participate in eosinophil recruitment. There were no statistically significant IL-13- or simvatatin-mediated effects on Th1/Th2 cytokines CCL12 (MCP-5) and TGFβ, or on the T_reg_ cytokine IL-10; all important players in asthmatic inflammation [46]. However, there was a trend of TGFβ inhibition by simvastatin, and given the role this cytokine plays in asthma, further research may reveal an important statin effect in epithelial cells.

In this study, simvastatin inhibited the expression of IL-13-inducible pro-inflammatory genes pertinent to asthma, however, it also unexpectedly induced the expression of some genes potentially relevant to inflammation, immune function, and cell survival. Whether statins affect gene expression at the transcriptional or post-transcriptional level in airway epithelial cells, and the mechanisms involved, remains to be determined. We believe it is important to report these statin effects on murine airway epithelial cells in order to refine future work in this field, at least as relevant to mouse models that utilize the airway epithelium. Furthermore, this *in vitro* ALI/biphasic model is an efficient and experimentally accessible cell culture system that mimics ‘near-physiological’ conditions and encourages the testing of a wider range of statin doses on airway epithelial function in general [49,50]. This facilitates the direct study of statins’ molecular mechanisms of action, as compared to less direct animal models with the attendant complexities of the *in vivo* environment.

We also chose the MTE cell culture system because it is a natural extension of our prior work in the ovalbumin allergic mouse model. We wanted to know the relative contribution of the murine epithelium to allergic airway inflammation, and whether simvastatin has direct beneficial effects on this key constituent of the airway. The MTE system allowed us to study the statin effect *in isolation* without the numerous cell-cell interactions that can limit data interpretation and the complex influence of leukocyte-epithelial interactions that happen *in vivo.* What we learn from MTE cells can be applied to ongoing studies using human airway epithelial cells, as knowledge gained from one approach will inform the other.

We are not aware of any studies which evaluate the effect of statins on airway epithelial gene expression using microchip arrays. This study is a small step in that direction and gives a broader picture of which airway epithelial genes are up- and down-regulated by statins. However, given that we do not know the full spectrum of the statin effect on airway epithelial cell function, additional studies aimed at uncovering the physiological consequences and signaling pathways involved are warranted. At the dose and treatment duration examined in our study, simvatatin clearly has differential effects on pro-inflammatory epithelial gene expression. Thus, additional dose- and time-response studies, and the screening of both hydrophilic and hydrophobic statins, may shed light on the spectrum of statin effects on epithelial gene expression. Furthermore, whether or not simvastatin’s effects on cytokine gene expression involves HMGR inhibition, the canonical target of the statin drugs in the MA pathway, is a hypothesis worth testing.

Whether or not our findings can be extrapolated to human airway epithelial cells is not entirely clear. However, the basic mechanisms related to mevalonate and HMGR biology are generally conserved across eukaryotes which suggest that the statin effects should also be similar. Although not studied directly in asthmatic airways or under allergic conditions, the few published papers evaluating the effects of statins on human bronchial epithelial cells show inhibition of pro-inflammatory cytokine/chemokine expression [15,16,21]. This leads us to believe that our results may be extrapolated to human epithelial cells, although more research is needed to further test this hypothesis and the underlying mechanisms of action.

There are several limitations to the work presented here: (1) We evaluated a relatively small number of IL-13-inducible mouse genes relevant to allergic asthma. Indeed, the statins may be affecting a host of other genes not evaluated in our experiments. Because the pathogenesis of allergic asthma is complex and polygenic, a more complete evaluation of statins’ effects on lung gene expression is needed. (2) We did not determine the mechanism(s) of how simvastatin inhibits gene expression, however, we speculate that the HMGR enzyme/MA pathway plays an important role. (3) Because this was a mouse *in vitro* study, we cannot necessarily generalize to the *in vivo* situation, and extrapolation to human disease would be premature. (4) Inhibition of gene expression in the airway epithelium will not necessarily translate into clinical benefits, but such findings are promising. In future studies, evaluating human tracheobronchial epithelial brush biopsies from patients taking statins could more directly establish translational relevance.

## Conclusions

Our data indicate that simvastatin modulates murine airway epithelial inflammatory gene expression relevant to asthma. In addition to inhibition of IL-13-inducible cytokines, simvastatin also induced some genes relevant to inflammation and host defense. The statins may have a therapeutic role in asthma or COPD, however, more work is needed to characterize the mechanism(s) of how statins affect gene expression in airway epithelial cells. Similar evaluations in human airway epithelial cells derived from patients with asthma and COPD are necessary and will guide future clinical studies.

## Abbreviations

MA: Mevalonate; HMGR: HMG-CoA reductase; MCP: Monocyte chemotactic protein; ALI: Air-liquid interface; Sim: Simvastatin; SPP1: Secreted phosphoprotein 1; FPP: Farnesylpyrophosphate; GGPP: Geranylgeranylpyrophosphate; COPD: Chronic obstructive pulmonary disease; GTPase: Guanosine triphosphatase; i.p.: Intraperitoneal; NK: Natural killer cells; FEV1: Forced expiratory volume in the first second; FVC: Forced vital capacity; AHR: Airway hyperreactivity; PE: Polyethylene; RA: Retinoic acid; DMSO: Dimethyl sulfoxide; Ct: Threshold cycle; GOI: Gene of interest; SEM: Standard error of the mean; ANOVA: Analysis of variance; RT-PCR: Quantitative real time-polymerase chain reaction; CC: Cysteine-cysteine; CCL: Cysteine-cysteine ligand; NK: Natural killer; HBD-2: Human β-defensin-2; MTE: Mouse tracheal epithelium; Treg: Regulatory T-cell.

## Competing interests

The authors declare that they have no competing interests.

## Authors’ contributions

RW and AAZ conceived of the project, and experimental design. PT helped refine the experimental approach and assisted with RT-PCR. AAZ carried out the tracheal harvesting, cDNA preparation, and running the RT-PCR arrays. AAZ did the statistical analysis. NJK had significant intellectual input into the development of this work, and added to the Discussion. All authors reviewed data and results, and had significant input into the writing of the final manuscript. All authors read and approved the final manuscript.
